# The Use of the Artificial Membrana Tympani

**Published:** 1866-04

**Authors:** D. B. St. John Roosa

**Affiliations:** Aural Surgeon to the New York Eye and Ear Infirmary. Lecturer in the University of the City of New York


### THE USE OF THE ARTIFICIAL MEMBRANA TYMPANI.

By D. B. St. JOHN ROOSA, M.D., Aural Surgeon to the New York Eye and Ear Infirmary. Lecturer in the University of the City of New York.

Case I. A farmer, *æt.* 30, from Michigan (at the Infirmary), January, 1865. The patient had scarlet fever 13 years ago, since which time he has suffered from periodical attacks of pain referred to the ears, discharge of pus from them, and vertigo. He has also been so deaf as not to hear ordinary conversation ever since the attack of scarlatina. Patient's general condition is bad, he having suffered much from intermittent fever. Cannot hear a watch, which should be heard by a person with normal hearing power, more than four feet, at all, neither on auricle, mastoid process, nor frontal bone. The right membrana tympani has been wholly removed by ulceration, no trace of *ossicula auditus.* Mucous membrane of the cavity of the tympanum hypertrophied. A portion of the periphery is all that remains of the left membrana tympani. The incus and stapes remain in situ, but the malleus has been lost. Mucous membrane of cavity of the tympanum also hypertrophied. Both Eustachian tubes are pervious, as proven by the Valsalvian experiment. The artificial membrana tympani was placed in the right ear without producing the slightest benefit; being inserted in the left, it immediately so improved the hearing, that the watch could be heard two inches from the auricle, and ordinary conversation several feet. The patient was enabled to pronounce isolated words after a speaker standing more than 12 feet distant. The patient was under observation for a few days, during which time the hearing remained as good as above stated. He then left for his home, taking with him a supply of the artificial membranes.

Case II. Miss. U., *æt.* 30, N.Y., May 31, 1863. Patient has been deaf ever since she can remember. Does not hear conversation unless specially addressed, and then the voice must be raised. She knows no cause for the deafness. Hears the watch two inches from the right auricle, not at all on the left side, except upon the mastoid process. Left membrana tympani opaque in its mucous and fibrous layers. The light spot is lessened in size, and the head of the malleus is abnormally prominent. Right membrana tympani perforated by ulceration in centre, the remaining portion is granulated. A very slight amount of greenish fœtid pus is secreted by the cavity of the tympanum and the remains of the drum. The pharynx is congested. Eustachian tubes impervious, as shown by the Valsalvian experiments, Politzer's method, and the catheter. General health not good, although no especial disease is recognized. Patient was seen every few days until August 5th, during which time the following treatment was carried on:—Permeability of the Eustachian tubes was secured by the use of the catheter and Politzer's method, together with the use of gargles, and a weak solution of sulphate of zinc (gr. j. ad. aq. ℥j.) was applied to the drum, after daily syringing, in order to check the ulcerative process. When this was restrained, an artificial membrana tympani was applied and worn except at night. It caused, at first, much irritation and furuncular inflammation. The artificial drum was removed until this was checked. The drum is now worn all day, and the watch is heard from six to eight inches with it, only two without it. Ordinary conversation heard fairly; hearing on the other side as before. Patient expresses herself as being very much improved.

Case III. J. J. V. P., *æt.* 28, La., Aug. 12, 1865. Three years ago, while in the artillery service, patient lost his hearing gradually, although he remembers at one particular time, after being engaged in heavy firing, that he had a distinct sensation of ringing and fulness in his ears. When a child he had the same sensation in the right ear, after which he was deaf from that ear for some time. The ears were treated by the medical officer of the regiment by the application of tannic acid. He continued in the service until the end of the war, and was subjected to various kinds of treatment, application of arg. nit., cup. sulph., and other astringents. At times, he could hear quite well, and then his ears were "stopped up" for a time. He was exposed to much hardship during a great part of his term of service. The deafness has increased until now, when he cannot hear at all from the right ear, and from the left with the aid of an ear trumpet. He does not hear the watch at all on either side. The right membrana tympani, except as to the upper portion, where a small rim remains, has been removed by ulceration. The integument of the auditory canal, and the mucous membrane of the cavity of the tympanum are hyperæmic and swollen. The little bones of hearing cannot be found. There is a slight amount of fœtid pus secreted by the mucous membrane. On the left side, the auditory canal is extremely hyperæmic, swollen, and tender. The epidermis is exfoliating. The membrana tympani is not seen, but the Valsalvian experiment shows that it is perforated. Both Eustachian tubes are open.

*October* 18. Since the first date, the patient has been seen twice a-week, and has been treated in the following manner:—The ears have been gently syringed with warm water twice a-day, a weak solution of the sulphate of zinc (gr. ss. ad. ℥j.) has been dropped into the auditory canal and cavity of the tympanum, always warming it before use, and injections of the vapor of iodine have been made into the middle ear, by means of the combination of Politzer's method for rendering pervious the Eustachian tube with an inhaler, described by Dr. Buttles of this city.* (This method of combination is, I believe, original with myself. Buttles' inhaler consists, essentially, of a hollow buld of hard rubber with a nozzle. In the cavity of the bulb is placed a small sponge, which is saturated with the tincture of iodine. This bulb is attached to a bit of rubber tubing, and this is in turn placed over the pipe of an ordinary soft rubber globular syringe (Politzer). The nozzle of the inhaler is inserted in one nostril, the other being closed with the finger, the mouth is also shut, and the patient told to swallow (a little water facilitates this); just as the patient is in the act of swallowing, the physician compresses the bulb of the syringe, the Eustachian tubes open, and the air, iodized, passes into the cavity of the tympanum, or, barring a mooted point, into the faucial orifice of the Eustachian tube.)

The condition of the patient's ears is now as follows:—On the right side the hyperæmia and swelling are reduced to a minimum, as also on the left. In the left cavity of the tympanum the incus in position can now be distinctly defined. On this side the artificial drum is worn by day, except when the patient is alone for some hours, and removed at night. On the right side the drum has been worn at times, but never with any appreciable change as to the hearing power, which remains as when patient first came under observation, except that he can now hear the alphabet pronounced through an elastic tube.

On the left side he can hear the watch over the auricle, and ordinary conversation near at hand with ease. He can hear a sermon in church, and goes once more into society, from which his previous amount of deafness completely shut him out. He does not now use an ear trumpet at all, hearing better without

* New York Medical Journal, July, 1866.

the drum than he did formerly with the aid of a conductor of sounds. The patient is extremely intelligent, and to his strict attention to the directions given—his careful use of the artificial drum, removing it whenever it has caused the slightest irritation—a great part of the modicum of success attained is due.

CASE IV. Miss N., *æt.* 18, July 18, 1865. One year ago was quite ill, the nature of the affection cannot now be accurately ascertained. During the sickness both ears began to discharge pus, and deafness appeared. The discharge was checked, but the deafness has gradually increased until now, when she cannot hear ordinary conversation, and hears the watch tick only one inch from the auricle. Each membrana tympani has a central perforation, and there is a slight amount of yellow fœtid pus secreted in the cavity of the tympanum. The Eustachian tubes are pervious. The artificial drum improves the hearing on each side, by the watch, to a distance of six inches, and renders ordinary conversation easily heard. The patient was directed to daily syringe the ears with tepid water, using afterwards an astringent, and to wear the drum during the day. Patient has come to the office very irregularly, and carried out the directions very inefficiently. She seems to have an aversion to the use of the drums, wishes to be cured without wearing them. They cause very considerable irritation of the auditory canal.

CASE V. Rachel C., *æt.* 16, April 1, 1865, (at the Infirmary.) One year and a-half ago, patient discovered that she did not hear well. The deafness still continues, with some occasional pain and noise in the ears. She can hear the watch three inches from the right ear, one inch from the left. There is a perforation of each drum, with a slight ulcerative process going on in the membrane. Patient is of a strumous diathesis, has a curvature of the spine, but is just now in fair general health. Careful syringing of the ears, followed by the use of an astringent, was directed. There is no account of the condition of the Eustachian tubes until June 8, when Dr. C. E. Hackley inserted an artificial membrana tympani and made the following note:—"Artificial drum tried on the right side, of which the Eustachian tube is pervious, the hearing distance increased to ten inches. Politzer's method of rendering pervious the tube was practised.

*June* 22. "Left Eustachian tube is now pervious, with artificial drum, hearing advanced to twelve inches."

*Sept.* 14. I saw the patient and made the following "Patient has been in the country and has worn the artificial drum by day ever since. Hearing distance, right ear, twenty inches, left two feet. Drums cause no irritation whatever."

*Remarks.*—Surgical literature, so far as I can find, has comparatively little reference to cases in which the artificial drum has been worn. I have, therefore, given the foregoing somewhat in detail, in order to show about what may be accomplished by the substitute for the natural membrane. The cases have been taken, without any particular choice, from a number of which I have notes. It is the habit of the writer to tentatively apply the artificial membrane to all ancient perforations, where the hyperæmia and inflammation or discharge of pus are not very considerable. Recent cases of perforation, as a rule, heal so readily that the use of the drum is not indicated.

In order to a successful use of the artificial membrane—

1. The Eustachian tube must be pervious.
2. The stapes or incus of the ossicula must be *in situ.*
3. The inflammatory action in the external auditory canal and remains of the drum must not be excessive.

It is also of great assistance to the surgeon in procuring a successful wearing of it, that the patient should be intelligent enough to realize that at the best the disc of rubber is a foreign body, which should be carefully removed at any approach of irritation. It is, therefore, not of much use in the case of children, or unusually stupid or careless adults. It should also be stated, that cases have been found where all the above-named conditions have been fulfilled, where it was *a priori* supposed that the artificial membrane would do good, and yet repeated trials proved that the use of it effected nothing for the hearing. In these cases we may perhaps conclude that there existed very considerable rigidity of the quasi articulation of the stapes with the *fenestra ovalis.—American Jour. Med. Sciences.*

